# Inappropriate Patient Sexual Behaviour Towards Female Chiropractors in South Africa: A Quantitative Analysis

**DOI:** 10.1016/j.echu.2025.07.006

**Published:** 2025-09-15

**Authors:** Christopher Yelverton, Kamilah Jordaan

**Affiliations:** Department of Chiropractic, Faculty of Health Sciences, University of Johannesburg, Johannesburg, Gauteng, South Africa

**Keywords:** Female, chiropractors, sexual harassment, workplace

## Abstract

**Objectives:**

The purpose of this study was to survey chiropractors’ awareness and use of inappropriate patient sexual behavior (IPSB) protocol guidelines.

**Method:**

This study used a 31-question online survey, distributed during the period of September to November 2022, to all registered female chiropractors in South Africa. The survey focused on how often and how severe IPSB incidents were, as well as practitioners’ knowledge and use of IPSB guidelines.

**Results:**

Of the 508 chiropractors, 24.2% (123) responded. All respondents were aware of IPSB, with 59.8% reporting they had experienced it. More than half (52.2%) had encountered IPSB more than once, with mild cases being the most common, followed by moderate and severe cases. Respondents reported they felt more prepared for mild IPSB than for moderate or severe incidents. Many (40.9%) chose to ignore the behavior and continue treating the patient, while 34.1% gave a verbal warning before continuing care. A majority (95.7%) were concerned about IPSB, and 82.9% thought additional training could help them better handle these situations. While 62.6% knew about the IPSB protocol guidelines, 80.5% had not read them. Nevertheless, 83.3% said they had implemented the guidelines, and 66.7% found them helpful for educating chiropractors about IPSB.

**Conclusion:**

The study found that 59.8% of female chiropractors in South Africa have experienced IPSB. Maintaining professional boundaries is a major concern for this group of chiropractors, and further research and professional guidelines may be necessary to address the impact comprehensively.

## Introduction

Sexual harassment and assault of women in the workplace is an international concern. In the United States, 81% of women reported experiencing sexual harassment and/or assault in their lifetime and 38% of women experienced sexual harassment in the workplace.^1^ These events have been shown to have a substantial impact on both physical and psychological health.^2^

Inappropriate patient sexual behavior (IPSB) is defined as any verbal or physical act of an explicit, or perceived, sexual nature, which is unacceptable within the social context in which it is carried out.^3^^,^^4^ This can be linked to gender-based violence (GBV) as both involve the abuse of power, control, or dominance, often disproportionately targeting women and perpetuating harmful gender norms and dynamics in healthcare settings. In South Africa, up to half of women have been estimated to encounter gender-based and/or sexual violence from a partner during their lifetime.^5^ he impact of IPSB includes psychological stress, negative work-related consequences, career dissatisfaction, and burnout among practitioners.^6^^,^^7^

A healthcare provider/patient relationship depends on trust and confidence.^8^ Explicit professional boundaries need to be in place to assist in avoiding patients who act sexually or approach healthcare providers in an unwelcome or uninvited manner.^8^^,^^9^ Women in the healthcare workplace have been reported to be more empathic and caring towards their patients, ask more psychosocial questions and spend more time with their patients, which may make them more vulnerable to IPSB.^10^ Boundary violations may include unsolicited sexual conversations, derogatory noises such as “cat calls”, suggestive jokes, indecent exposure, kissing, embracing, or the patient touching their genitals in front of the health care provider.^6^ Sexual behaviour can be described as any acts, words, conduct/behaviour and inappropriate or unwelcome gestures designed or intended to arouse or gratify sexual impulses or desires.^11^ Inappropriate patient sexual behaviour (IPSB) is described as sexual harassment by patients directed towards a healthcare professional.^12^

Healthcare professionals may be more vulnerable to sexual harassment by patients due to elements of professional interactions such as a face- to-face interaction in a private setting, the potential perceived power difference between the practitioner and their patient, and the idea that medical professionals are trained to prioritise the patient’s needs over their own.^13^ Sexual remarks and remarks about physical appearance are the most common form of IPSB recorded^13^, ^14^, ^15^, ^16^, ^17^ (Supplementary file, Table 1).

**Table 1:** Summary of the participants’ ages

Age range	% (n)
25 years and younger	6.5 (8)
26-35 years old	59.3 (73)
36-45 years old	26.8 (33)
46-55 years old	6.5 (8)
56-65 years old	0.8 (1)

Hands-on care and physical touch are performed by manual therapists such as physiotherapists, chiropractors, and osteopaths during therapeutic interventions. The nature of the interventions therefore has the potential to increase susceptibility of fthese allied health professions to IPSB ^7^. The prevalence of IPSB for manual therapists ranges from 42.1% to 86.1% .^6^^,^^18^, ^19^, ^20^, ^21^ (Supplementary file Table 2) Sexual remarks and offensive jokes/suggestive stories were the most common IPSB displayed. Female chiropractors have reported being sexually harassed by patients, with incidents commonly occurring within the first 5 years of practice and involving new patients.^6^

**Table 2:** Awareness, frequency, frequency in 12 months and age of patient involved with incident of IPSB

Awareness of IPSB	% (n)
Slightly aware	1.6 (2)
Somewhat aware	17.2 (21)
Moderately aware	41 (50)
Extremely aware	40.2 (49)
**Frequency of IPSB**	
About once a week	1.4 (1)
About once a month	4.3 (3)
About once in three months	4.3 (3)
About once in six months	10.1 (7)
About once in the past year	7.2 (5)
It was a once off event	20.3 (14)
It happened a few times at random intervals	52.2 (36)
**Frequency of IPSB in 12 months**	
One time	21.4 (15)
2-3 times	44.3 (31)
4-5 times	21.4 (15)
6-10 times	7.1 (5)
11-15 times	1.4 (1)
More than 15 times	4.3 (3)
**Age of patients involved in incident of IPSB**	
Below 25 years	5.5 (7)
26-35 years	14.8 (19)
36-45 years	28.1 (36)
46-55 years	28.1 (36)
56-65 years	16.4 (21)
Above 65 years	7 (9)

To assist South African chiropractors in managing IPSB, the Chiropractic Association of South Africa (CASA) and Chirosport South Africa (SA) created a protocol guideline entitled “Creating and Maintaining Clear Sexual Boundaries Between Chiropractor and Patient”.^11^ These guidelines aimed to highlight the responsibilities of chiropractors to establish and maintain clear sexual boundaries with patients and the processes to follow should these boundaries be crossed.

The current literature on sexual harassment within the workplace and, more specifically IPSB amongst health care professionals, suggest a high prevalence more specifically toward women.^6^^,^^13^, ^14^, ^15^, ^16^, ^17^, ^18^, ^19^, ^20^, ^21^ In South Africa, approximately 54.8% of chiropractors are female, with an increasing trend.^22^^,^^23^ Therefore the purpose of this study was to survey chiropractors’ awareness and use of inappropriate patient sexual behavior (IPSB) protocol guidelines. In addition, current IPSB guideline protocols were evaluated in relation to awareness and application.

## Method

### Study design

This explorative, quantitative, and descriptive study employs a non-probability, voluntary response sampling method that adheres to the CHERRIES (Checklist for Reporting Results of Internet E-Surveys) guidelines for reporting.^24^^,^^25^ The survey was administered as an anonymous online questionnaire targeting female chiropractors registered with the Allied Health Professions Council of South Africa (AHPCSA).

### Participants and recruitment

The target population consisted of 508 registered female chiropractors in South Africa. The sample size was 115 valid responses based on an 8% margin of error and a 95% confidence interval. The chosen margin of error of 8% strikes a balance between precision and feasibility, providing a reasonable range within which the true population parameter is expected to lie while avoiding the need for a prohibitively large sample size. The 95% confidence interval is a widely accepted standard in research, offering a high level of certainty that the true population parameter falls within the estimated range without being excessively stringent. This sample size ensures statistical significance and reliable inferences while remaining practical and manageable within the study’s logistical constraints.^26^

Participants were recruited through multiple channels to ensure broad reach. Email sent by the AHPCSA and the Chiropractic Association of South Africa (CASA) included the survey link and a QR code was displayed at the Chiropractic Association of South Africa’s Congress in September 2022. Participation was voluntary, with no incentives offered, and responses were collected over a 2month period from 16 September 2022 until 16 November 2022.

### Inclusion and Exclusion Criteria

Eligibility was restricted to female chiropractors, determined by a screening question at the start of the survey asking participants to confirm their sex. Male chiropractors were automatically excluded based on this criterion.

### Survey Instrument

The survey was adapted from previous study by Innes et al. (2021)^7^, exploring the IPSB experiences of Australian female chiropractors.^7^ The survey was developed and modified with some questions adapted from additional studies.^6^ Statisticians from the university facility (STATKON) assisted with this process, and the survey underwent pilot testing with 5 academic staff members from the Department of Chiropractic at the University of Johannesburg to ensure clarity, relevance, and reliability of the questions.

The survey was composed of 4 sections (A-D) to systematically gather data on demographic details, awareness of IPSB guidelines, and experiences with inappropriate patient sexual behavior. See supplementary file for full survey.

Section A was a single screening question, namely “Sex?”. This allowed only participants who met the inclusion criteria to complete the survey. Section B was general information and biographical data. Questions 2-7 gathered information such as relationship status, region of primary practice, current age, main focus of chiropractic care, total number of employees and average number of patient visits in a week. Section C focused on the CASA and Chirosport South Africa’s IPSB guideline protocol for creating sexual boundaries between chiropractors and patients. Questions 8-12 gathered information on participants' awareness of the protocol guideline and whether they have read and implemented it. Question 13 focused on the awareness of IPSB amongst female chiropractors, and question 14 asked whether they have ever experienced this type of behaviour. When the participant answered “No” to question 14, they were directed to question 29 in section D. The participants who answered “Yes” in question 14 progressed to section D, question 15. Section D is related to the preparedness, prevalence, and extent of IPSB. Questions 15-18 gathered information about the prevalence, gender and age of the patient who displayed this type of behaviour. Questions 19-28 were adapted from Boissonnault et al. (2017)^21^ and investigated the severity of the IPSB. The behaviour was placed in three categories: mild, moderate and severe.^21^^,^^27^ The "mild" category was composed of questions related to suggestive stories or offensive jokes, sexual remarks about the therapist's appearance, uncomfortable staring or leering, and asking for a date. "Moderate" IPSB included crude sexual remarks, discussions about the therapist's private sexual matters, propositions for sexual intercourse, and mild inappropriate touching. The "severe" category consisted of questions pertaining to deliberate sexual exposure, fondling, forceful touching, attempted rape, and rape (see supplementary file Table 3).^21^^,^^27^ Questions 29-31 were adapted from Gleberzon, et al.^6^ and related to female chiropractors' preparedness for an IPSB event and whether they thought additional training might be valuable.^6^

**Table 3:** Summary of frequency of examples of mild, moderate and severe IPSB

Mild IPSB	% (n)
None of the above	7 (5)
At least 1 of the above	18.3 (13)
Two or more of the above	33.8 (24)
Three or more of the above	29.6 (21)
All of the above	11.3 (8)
**Moderate IPSB**	
None of the above	19.7 (14)
At least 1 of the above	45.1 (32)
Two or more of the above	19.7 (14)
Three or more of the above	12.7 (9)
Four or more of the above	1.4 (1)
All of the above	1.4 (1)
**Severe IPSB**	
None of the above	73.2 (52)
At least 1 of the above	21.1 (52)
Two or more of the above	4.2 (3)
Three or more of the above	1.4 (1)

### Data Collection and Privacy Measures

Data were collected anonymously via an online survey platform to protect participant privacy. Measures were implemented to prevent duplicate entries, such as IP address checks. Data were stored securely and were only accessible to authorized research team members to maintain confidentiality.

### Handling Non-Response and Missing Data

The survey was designed with mandatory questions where appropriate, while allowing participants to skip questions related to sensitive experiences to minimize distress. Missing data were handled by directing non-respondents of critical questions to subsequent relevant sections without penalty, ensuring ethical management of sensitive information.

### Statistical Analysis

Statistical analysis was performed using SPSS Statistical Package Version 28 (IBM, Armonk, NY, USA). Cross tabulations were used to compare categorical variables and Chi-square tests, including Fisher’s Exact Test were applied to evaluate the significance of observed differences, with a significance level set at p<.05.

### Ethical Considerations

The research was approved by the University of Johannesburg, Faculty of Health Sciences Research Ethics Committee (REC-1708-2022). The participants were informed that their participation was voluntary and confidential, and they were allowed to withdraw from the study at any point before submitting the questionnaire.

This survey questioned sensitive information, and participation was anonymous and confidential to protect the participants. No identifying data was collected, and responses could not be traced back to the participant. By completing this survey, there was a risk that participants may recall sensitive or traumatic experiences which may have been emotionally distressing after reading and answering the survey questions. A mental health helpline, depression and anxiety helpline, gender-based violence (GBV), and suicide crisis helpline were made available.

## Results

Of the 508 potential participants, 24.2% (n=123) valid responses were obtained. Gauteng was the predominant region, with 61.5% (n=75) indicating this region as their primary practice. With the majority of the responses (58.8%, n=72) indicated participants were married and between the ages of 26 and 35 years old (59.3%, n=73) (Table 1). General musculoskeletal care was the predominant focus of chiropractic care (54.5%, n=67), with 52.8% (n=65) indicating they were the only person in a clinical setting (52.8%) with an average between 21 and 40 patients seen per week (42.3%, n=52).

### Inappropriate patient sexual behaviour

All participants were aware of IPSB (Table 2). The prevalence was 59.8% (n=73), with 52.2% (n=36) experiencing IPSB sporadically and 44.3% (n=31) reporting an incident in the past 12 months (Table 2). Most perpetrators were male (88.6%, n=62), while 10% (n=7) could be of either sex, and 1.4% (n=1) declined to specify. Patients were most frequently aged 36–45 (28.1%, n=36) or 46–55 (28.1%, n=36) (Table 2)

Participants were provided with examples of mild, moderate, and severe IPSB and asked to report their experiences. Most had encountered mild IPSB (93%, n=66) (Table 3). Unanticipated exposure was common, particularly for severe IPSB (94.7%, n=18) with preparedness decreasing with severity: 59.1% (n=39) felt adequately prepared for mild IPSB, whereas 52.6% (n=30) and 89.5% (n=17) reported inadequate preparedness for moderate and severe incidents, respectively.

When responding to IPSB, the most common action was ignoring the incident and continuing care (40.9%, n=36) Rare responses included contacting the regulatory body (AHPCSA; 1.1%, n=1) or taking other actions (2.3%, n=2).

Nearly all participants (95.7%) expressed some concern about IPSB, and 82.9% (n=58) believed additional training could have improved their ability to anticipate or manage such incidents

### Awareness and application of the IPSB protocol guide

Most participants (80.5%, n=99) had not read the CASA and Chirosport SA IPSB protocol guide, and only 8.9% (n=11) were "extremely aware" of it. Among those who had read it (n=20), 83.3% implemented the guide, and 66.7% (n=16) found it adequately educational. Additionally, 82.9% (n=58) believed additional training would have better prepared them for IPSB incidents (Table 4)

**Table 4:** Summary of awareness and application of IPSB protocol guide

Awareness of protocol guide	%(n)
Not at all aware	37.4 (46)
Slightly aware	13 (16)
Somewhat aware	25.2 (31)
Moderately aware	15.4 (19)
Extremely aware	8.9 (11)
**Read the protocol**	
Yes	19.5 (24)
No	80.5 (99)
**Understood the protocol**	
Yes	95.8 (23)
No	4.2 (1)
**Guideline adequacy**	
Yes	66.7 (16)
No	33.3 (8)
**Implementation of guideline**	
Yes	83.3 (20)
No	16.7 (4)
**Additional training beneficial**	
Yes	82.9 (58)
No	17.1 (12)

No statistically significant findings were noted in the analysis of comparisons

### Proposed factors that would have prepared respondents for IPSB

**Figure 1:**
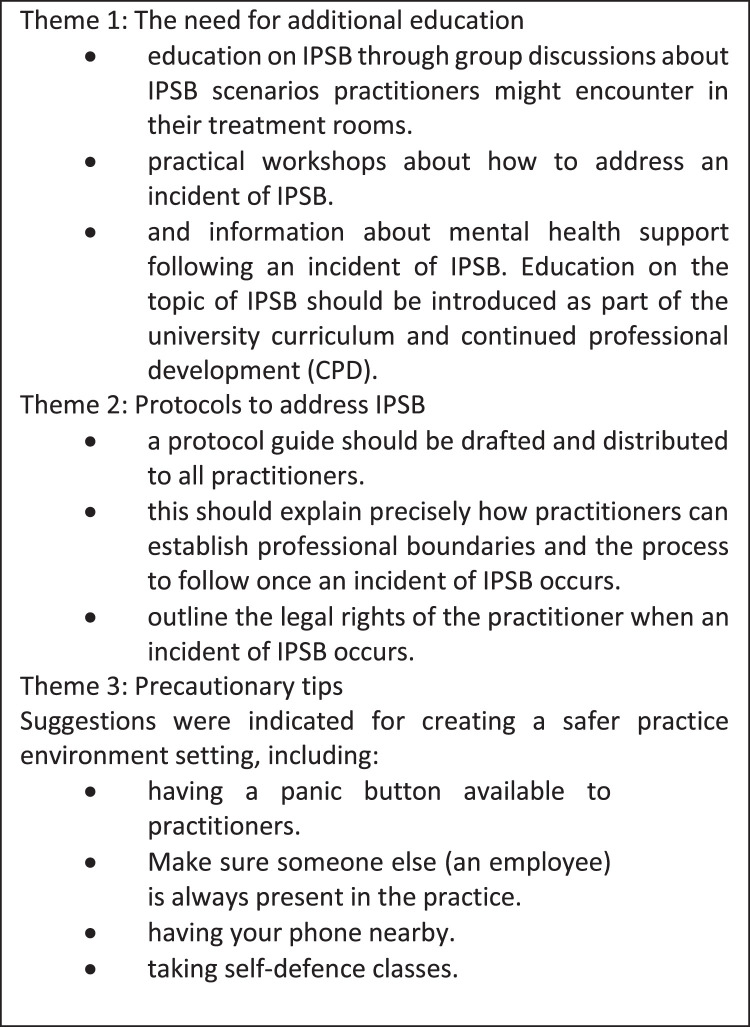
Themes from responses.

As an additional response, participants were asked to indicate what they thought could prepare them for any IPSB. Three themes were identified based on these responses and are shown in figure 1.

## Discussion

This study, which investigated the experiences of South African female chiropractors with IPSB, revealed a high prevalence of 59.8%, and suggests that mild IPSB was most prevalent, followed by moderate and severe. An incident of IPSB is generally not anticipated, especially in the case of a severe incident, where 94.7% indicated this was not anticipated and mild IPSB was better prepared for than moderate or severe levels. The most likely response from practitioners following an incident was to ignore it and continue care. A potentially aggravating factor was that the awareness of protocol guidelines was poor (34.7% awareness), but more concerning was the limited number that had actually read the document (19.5%). These documents appear not to be fit for purpose based on the findings, and suggestions in relation to this will be presented.

The response rate was comparable to previous studies in this topic,^6^^,^^19^^,^^20^ but was focused on female practitioners. The prevalence rate is similar to previous studies by Ang et al^28^ and Bütow-Dûtoit, et al^20^ who have reported figures of 58.2% and 52% respectively, however, this is lower than Weerakoon and O'Sullivan^19^ who reported 81.6%. The reported incidence rate of IPSB may be affected by the individual’s understanding of IPSB and what they perceive as sexual harassment from a patient. Weerakoon and O’Sullivan^19^ suggest this lack of perception may be due to the nature of the behaviour not being considered aggressive or hostile and the nature of chiropractic treatment involving physical contact. Due to the lack of clear guidelines on conduct within a chiropractic setting, the potential exists for personal interpretation and implementation of boundaries between practitioner and patient.

Severity levels of incidents correspond to previous studies^18^^,^^19^ who also indicated mild levels being the most common. Offensive jokes/suggestive stories, was the most common for (68%). Each person has a specific romantic relationship framework for assigning meaning to a specific event, which guides a person’s behaviour and emotional response to a specific event.^29^ For example, if a patient’s romantic relationship framework includes attentive listening and genuine kindness, two behaviours that chiropractors display, there is a risk that patients might develop romantic feelings for the practitioner. It is suggested that the IPSB may be initiated by developing romantic feelings for the practitioner and then escalating from mild to moderate and finally severe, should professional boundaries not be re-established following a single mild IPSB event.

The anticipation of an incident of IPSB was very low, with 94.7% indicating they did not, which is similar to previous studies.^6^ Practitioners may be less likely to anticipate IPSB when establishing clear professional boundaries between the patient and themselves. Participants may have felt they established adequate professional boundaries and did not therefore anticipate the incident. Preparation for a mild incident was most common in line with previous findings.^28^ In general, practitioners do not feel prepared to address IPSB, regardless of the severity of the behaviour displayed by patients, or their experience in practice.^7^

In our study and previous findings,^6^^,^^7^ participants indicated that the most utilized strategy to deal with an incident of IPSB was to ignore the event and continue care, which appears to be effective in the short term but may not prevent a future incident. The sensitive context of sexual behaviour, combined with practitioners not anticipating the incident, and not knowing how to address the matter with the patient, are potential factors for why this approach is adopted.

As supported by previous studies,^14^ almost all participants indicated they were, to some extent, concerned and given the high prevalence in our study, it may be expected that practitioners are concerned about the problem of sexual harassment from their patients. Most participants in our study and previous studies^20^^,^^28^ felt additional training could have helped anticipate or manage an incident of IPSB. No universal code of conduct for creating professional boundaries exists within the chiropractic profession in South Africa and additional training needs to be considered for practitioners. There is also a need to include greater patient awareness of appropriate behaviour, specific curriculum content, assertiveness training in undergraduate programs, and the creation of ethical guidelines for patient behaviour by regulatory bodies.

Awareness of the protocol guide was low (37.4%), and may be attributed to the fact that this is only available to members of CASA and Chirosport, and, therefore not available to all practitioners. The majority (80.5%) had not read the protocol guide.. Correlations performed between reading the protocol guide and the preparedness level of participants for mild, moderate and severe IPSB suggest that reading the CASA and Chirosport SA protocol guide may have assisted participants in being more prepared for mild IPSB, but not necessarily for moderate and severe IPSB.

The majority (66.7%) of participants who had read the protocol guide indicated that the document was adequate for educating chiropractors in managing IPSB, with 83.3% of participants who had read the protocol guide implementing this in their practice. This indicates that the content of the protocol guide is adequate in helping create professional boundaries between chiropractors and their patients. No correlation for between the implementation of the protocol guide and the preparedness level of participants for mild, moderate and severe IPSB none could be established. Results suggest that participants require education on how to address severe IPSB and that the protocol guide may not be sufficient to help prepare practitioners for severe IPSB. In addition, no correlation could be established adequacy of professional boundaries and reading the protocol, and less participants who had read the protocol guide indicated they need for additional training compared to those who had not read the protocol guide. The majority (82.9%) indicated that additional training could have assisted them in being better prepared for the IPSB incidents.

The findings relating to the IPSB documentation indicate that although it may be fit for the purpose, more effort is required to increase awareness of the availability and ensure additional training is conducted to assist practitioners in the overall implementation.

Inappropriate patient sexual behavior in healthcare settings is a multifaceted issue influenced by various factors, including the prevalence of GBV and its significant impact on patient care in South Africa. Addressing IPSB requires a comprehensive approach involving legal and ethical considerations, targeted interventions, and improved support systems to mitigate the impact of GBV on survivors in South African healthcare settings.

### RECOMMENDATIONS

Recommendations are presented in 2 aspects, the first being to consider how the profession may deal with IPSB and then in terms of future research related to IPSB in the South African context.

The following recommendations are proposed for implementation within the South African chiropractic context to facilitate improved preparation for potential IPSB:

1. A global IPSB protocol guide should be developed and made available to all registered chiropractors, highlighting the code of conduct, creating professional boundaries between practitioner and patient and protocols to follow should an incident of IPSB occur. This should include information related to the practitioner's ethics and legal rights.
2. Given the high prevalence, additional training and awareness within educational institutions and practitioners' curriculums should be developed.
3. IPSB awareness campaigns should be developed to highlight the issue of IPSB and available guidelines to assist practitioners. These documents should be clear in terms of protocols to follow should this occur, and guidelines that should be considered to assist in avoiding the occurrence. This should include elements of reassurance and support to practitioners who have experience with IPSB.

Future studies should consider conducting a similar study on South African male chiropractors, and in collaboration with other jurisdictions to investigate and explore international awareness. In addition, the prevalence and awareness of IPSB within the context of undergraduate chiropractic training is an important area for future research.

An evaluation of the current available IPSB protocols should be conducted regarding content and determine what areas need improvement. These should be aligned with available guidelines and regional legislation and include qualitative research integration (“tell me about your experiences regarding IPSB”). This would allow the insights gained by female chiropractors to be used to design protocols, with evidence supporting its benefits in preparing both students and practitioners.

### LIMITATIONS

The study had several notable limitations. First, the sensitive nature of the research topic may have contributed to a lower response rate, as some participants might have been hesitant to engage with the subject matter. Additionally, practitioners who had not experienced IPSB may not have viewed the study as relevant to their practice, further limiting participation. Second, the study exclusively focused on female chiropractors in South Africa, meaning the experiences of male chiropractors in the same region were not explored, potentially narrowing the scope of findings. Finally, establishing clear correlations proved challenging because certain questions were filtered, and a larger sample size might have provided more robust insights into whether meaningful relationships existed between variables.

## Conclusion

In conclusion, there appears to be a high prevalence of IPSB towards female chiropractors in South Africa. Practical strategies to address and prevent IPSB include educating students and practitioners about sexual harassment, assertiveness training, and specific response strategies. The psychological impacts of IPSB on both patients and female chiropractors are substantial, potentially contributing to burnout. Further research and professional guidelines may be necessary to address these aspects comprehensively.

## Funding Sources And Conflicts Of Interest

This research was funded via the Supervisor Linked Bursary from the University of Johannesburg. No conflicts of interest are noted for this study**.**

## Appendix Supplementary materials

Image, application 1
