# Supplementary material for: Inappropriate Patient Sexual Behaviour Towards Female Chiropractors in South Africa: A Quantitative Analysis
Source: J Chiropr Humanit. 2025 Sep 15;32:1–8. doi: 10.1016/j.echu.2025.07.006 (PMC12464598; doi:10.1016/j.echu.2025.07.006)
Supplement: Supplementary file 1 [file mmc1.docx]

**Inappropriate Patient Sexual Behaviour Towards Female Chiropractors in South Africa: A Quantitative Analysis**

**Contents of Supplementary File**

- **Supplementary Table 1:** Summary of characteristics of IPSB studies in healthcare
- **Supplementary Table 2:** Summary of characteristics of studies about IPSB in manual therapy
- **Supplementary Table 3:** Examples of mild, moderate and severe IPSB14^21,27^.
- **Survey**

| **Steps of action** | Reported incidents to the police (5%) | **-** | **-** | **-** | Terminated relationship with patient (6%) |
| --- | --- | --- | --- | --- | --- |
| **Most common IPSB**  **Table 1:** Summary of characteristics of IPSB studies in healthcare | Sexual remarks (59%) | Compliments about physical appearance (98%) | **-** | Comments about physical appearance (85%) | Verbal remarks about physical appearance (53%) |
| **Prevalence rate (%)** | 77% | 71.2%  (Amongst females) | 52.1%  (Amongst male and female) | 84%  (Amongst females) | 66%  (Amongst females) |
| **Gender of the study population** | Female | Male and female | Male and female | Male and female | Male and female |
| **Profession** | Physicians | Medical students and recent graduates | Emergency medicine residents | Dermatology | Medical students |
| **Country** | Canada | United States of America | United States of America | United States of America | United States of America |
| **Author and year** | Philips & Schneider ^14^ | Schulte & Kay ^15^ | Schnapp et al. ^16^ | Notaro et al. ^13^ | Mahurin et al. ^17^ |
| **Steps of action**  **Table 2:** Summary of characteristics of studies about IPSB in manual therapy. | - | **-** | **-** | **-** | Ignored incident and continued care (37.5%) |
| **Most common IPSB (%)** | Sexual remarks (67%) | Offensive jokes/suggestive stories (68%) | Requesting physical contact (15.74%) | Mild IPSB | Sexual remarks (100%) |
| **Prevalence rate (%)** | 81.5% (Amongst male and female) | 86.1%  (Amongst females) | 52.%  (Amongst male and female) | 84% | 42.1% |
| **Gender of the study population** | Male and female | Male and female | Male and female | Male and female | Female |
| **Profession** | Physical therapist | Physiotherapist | Physiotherapist | Physical therapist clinicians and students | Chiropractor |
| **Country** | United States of America | Australia | South Africa | United States of America | Canada |
| **Author and year** | de Mayo ^18^ | Weerakoon & O’Sullivan ^19^ | Bütow-Dûtoit, et al. ^20^ | Boissonnault et al. ^21^ | Gleberzon et al. ^6^ |

**Table 3:** Examples of mild, moderate and severe IPSB^21,27^.

| **Mild IPSB** | **Moderate IPSB** | **Severe IPSB** |
| --- | --- | --- |
| a) The patient stared at you or your body parts in a way that made you uncomfortable. | a) The patient made an overtly sexual remark or joke. | a) The patient masturbated during a session. |
| b) The patient asked you out on a date. | b) The patient asked you questions about or commented on your sex life. | b) The patient deliberately exposed his or her genitals or breasts to you. |
| c) The patient gave you a romantic or sexual gift. | c) The patient shared a sexual fantasy about you. | c) The patient purposefully touched or grabbed you in a private area (thighs, genitals, breasts). |
| d) The patient made a sexually flattering or suggestive remark about you. | d) The patient made sexually suggestive gestures. | d) The patient repeatedly followed, watched, or harassed you inside or outside the workplace. |
|  | e) The patient propositioned you for sexual activity. | e) The patient threatened to force you or attempted to force you to submit to sexual activity. |
|  |  | f) The patient forced or coerced you to submit to sexual activity. |

**SURVEY**

**
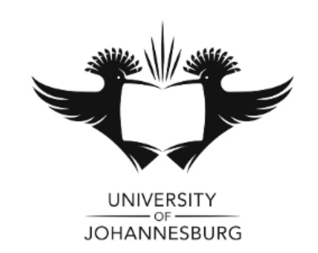
**

DEPARTMENT OF CHIROPRACTIC

RESEARCH STUDY: SURVEY

**SECTION A**

**Screening Question**

**1.Sex**:

| Male | 1 |
| --- | --- |
| Female | 2 |

*Answer specific, if the participant does not select "female", the survey will automatically go to the "thank you" page at the end as they do not meet the inclusion criteria.*

**SECTION B**

**General information and Biographical Data**

**2.In which province is your primary practice?**

| Gauteng | 1 |
| --- | --- |
| Kwa-Zulu Natal | 2 |
| Mpumalanga | 3 |
| Limpopo | 4 |
| North West | 5 |
| Eastern Cape | 6 |
| Northern Cape | 7 |
| Free State | 8 |
| Western Cape | 9 |

**3.Relationship Status** **[check one box only]**

| Single | 1 |
| --- | --- |
| Married | 2 |
| Divorced | 3 |
| Widowed | 4 |

**4.What is your current age?**

| 25 years and younger | 1 |
| --- | --- |
| 26 – 35 years old | 2 |
| 36 – 45 years old | 3 |
| 46 – 55 years old | 4 |
| 56 – 65 years old | 5 |
| Older than 65 years | 6 |

**5. What is the main focus of your chiropractic care?**

| Pediatrics and family care | 1 |
| --- | --- |
| Sports enthusiasts and athletes | 2 |
| General musculoskeletal care (spine and extremities | 3 |

**6.Total number of employees in clinical setting [check one box only]**

| One | 1 |
| --- | --- |
| Two | 2 |
| Three | 3 |
| Four | 4 |
| Five | 5 |
| Six | 6 |
| Seven or more | 7 |

**7.What is the average number of patient visits you (not clinic) see weekly?**

| 20 or less | 1 |
| --- | --- |
| 21 - 40 | 2 |
| 41 - 60 | 3 |
| 61 - 80 | 4 |
| 81 -100 | 5 |
| More than 100 | 6 |

**SECTION C**

**Chiropractic Association of South Africa and Chirosport South Africa’s protocol guide**

**8. Are you aware of the Chiropractic Association of South Africa and Chirosport South Africa’s protocol guide on creating sexual boundaries between chiropractor and patient?**

| Not at all aware | 1 |
| --- | --- |
| Slightly aware | 2 |
| Somewhat aware | 3 |
| Moderately aware | 4 |
| Extremely aware | 5 |

**9.Have you read the Chiropractic Association of South Africa and Chirosport South Africa’s protocol guide on creating sexual boundaries between chiropractor and patient?**

| Yes | 1 |
| --- | --- |
| No | 2 |

**If you have read the protocol guideline, please answer questions 10, 11 and 12. Otherwise skip to Question 13.**

**10. If you have read the protocol guideline, do you believe the Chiropractic Association of South Africa and Chirosport South Africa’s protocol guide is easy to understand?**

| Yes | 1 |
| --- | --- |
| No | 2 |

**11. If you have read the protocol guideline, do you believe the Chiropractic Association of South Africa and Chirosport South Africa’s protocol guide is sufficient to educate female chiropractors on how to address inappropriate patient sexual behaviour?**

| Yes | 1 |
| --- | --- |
| No | 2 |

**12. If you have read the protocol guideline, have you implemented the Chiropractic Association of South Africa and Chirosport South Africa’s protocol guide on creating sexual boundaries between chiropractor and patient in a private practice setting?**

| Yes | 1 |
| --- | --- |
| No | 2 |

**Inappropriate patient sexual behaviour**

**13. Are you aware of what inappropriate patient sexual behaviour is?**

| Not at all aware | 1 |
| --- | --- |
| Slightly aware | 2 |
| Somewhat aware | 3 |
| Moderately aware | 4 |
| Extremely aware | 5 |

**What is inappropriate patient sexual behaviour?**

Inappropriate patient sexual behaviour is any unwelcomed sexual behaviour from a patient that intimidates, humiliates or offends a practitioner and violates the practitioner’s boundaries.

**14. Have you ever experienced inappropriate patient sexual behaviour in private practice?**

| Yes | 1 |
| --- | --- |
| No | 2 |

**SECTION D**

**In the case that you have experienced inappropriate patient sexual behavior, please answer the following questions.**

**15. On average, how often do you experience inappropriate patient sexual behaviour? [check one box only]**

| About once a week | 1 |
| --- | --- |
| About once in two weeks | 2 |
| About once a month | 3 |
| About once in three months | 4 |
| About once in six months | 5 |
| About once in the past year | 6 |
| It was a once-off event | 7 |
| It happened a few times, at random intervals | 8 |

**16. How many times have you experienced inappropriate patient sexual behaviour? [check one box only]**

| 0 times | 1 |
| --- | --- |
| 1 time | 2 |
| 2 – 3 times | 3 |
| 4 – 5 times | 4 |
| 6 – 10 times | 5 |
| 11 – 15 times | 6 |
| More than 15 times | 7 |

**17.What was the sex of the patient who displayed inappropriate sexual behaviour? [check one box only]**

| Do not wish to indicate | 1 |
| --- | --- |
| Male | 2 |
| Female | 3 |
| Both male and female | 4 |

**18. What was the approximate age of the patient who displayed inappropriate sexual behaviour? [Mark all applicable ages if you had multiple incidents.]**

| 25 years and younger | 1 |
| --- | --- |
| 26 – 35 years old | 2 |
| 36– 45 years old | 3 |
| 46 – 55 years old | 4 |
| 56 – 65 years old | 5 |
| Older than 65 years | 6 |

**19. How many of the following mild inappropriate patient sexual behaviours have you experienced?**

**a.) Patient stared at you or your body parts in a way that made you uncomfortable**

**b.) Patient asked you out on a date**

**c.) Patient gave you a romantic or sexual gift**

**d.) Patient made a sexually flattering or suggestive remark about you**

| None of the above (go to question 22) | 1 |
| --- | --- |
| At least 1 of the above | 2 |
| 2 or more of the above | 3 |
| 3 or more of the above | 4 |
| All 4 of the above | 5 |

**20. Did you anticipate the incident/s of mild inappropriate patient sexual behaviour?**

| Yes | 1 |
| --- | --- |
| No | 2 |

**21. Did you feel adequately prepared for the incident/s of mild inappropriate sexual behaviour?**

| Yes | 1 |
| --- | --- |
| No | 2 |

**22. How many of the following moderate inappropriate patient sexual behaviours have you experienced?**

**a.) Patient made an overtly sexual remark or joke,**

**b.) Patient asked you questions about or commented on your sex life**

**c.) Patient shared a sexual fantasy about you**

**d.) Patient made sexually suggestive gestures**

**e.) Patient propositioned you for sexual activity**

| None of the above (go to question 25) | 1 |
| --- | --- |
| At least 1 of the above | 2 |
| 2 or more of the above | 3 |
| 3 or more of the above | 4 |
| 4 or more of the above | 5 |
| All 5 of the above | 6 |

**23. Did you anticipate the incident/s of moderate inappropriate patient sexual behaviour?**

| Yes | 1 |
| --- | --- |
| No | 2 |

**24. Did you feel adequately prepared for the incident/s of moderate inappropriate sexual behaviour?**

| Yes | 1 |
| --- | --- |
| No | 2 |

**25. How many of the following severe inappropriate patient sexual behaviours have you experienced?**

**a.) Patient masturbated during a physical therapist session**

**b.) Patient deliberately exposed his or her genitals or breasts to you**

**c.) Patient purposefully touched or grabbed you in a private area (thighs, genitals, breasts) and/or in a clearly sexual manner**

**d.) Patient repeatedly followed, watched, or harassed you inside or outside the workplace**

**e.) Patient threatened to force you or attempted to force you to submit to sexual activity**

**f.) Patient forced or coerced you to submit to sexual activity**

| None of the above (go to question 28) | 1 |
| --- | --- |
| At least 1 of the above | 2 |
| 2 or more of the above | 3 |
| 3 or more of the above | 4 |
| 4 or more of the above | 5 |
| 5 or more of the above | 6 |
| All 6 of the above | 7 |

**26. Did you anticipate the incident/s of severe inappropriate patient sexual behaviour?**

| Yes | 1 |
| --- | --- |
| No | 2 |

**27. Did you feel adequately prepared for the incident/s of severe inappropriate sexual behaviour?**

| Yes | 1 |
| --- | --- |
| No | 2 |

**28. Following the incident/s, what was your first step of action? [Mark all applicable if you had multiple incidents.]**

| Ignored and continued care | 1 |
| --- | --- |
| Verbal warning and continued care | 2 |
| Ask patient to leave immediately | 3 |
| Ask patient to leave after attempted care | 4 |
| Legal action | 5 |
| Contacted governing body (AHPCSA) | 6 |
| Other (please specify) | 7 |

**29. Do you feel that additional training or preparation for such events provided through continuing education or time in university could have helped you anticipate or handle such an event?**

| Yes | 1 |
| --- | --- |
| No | 2 |

**30. How would you rate the severity of the problem of sexual harassment of practitioners by their patients?**

| Not at all concerned | 1 |
| --- | --- |
| Slightly concerned | 2 |
| Somewhat concerned | 3 |
| Moderately concerned | 4 |
| Very concerned | 5 |

**31. What do you think would prepare you better for any type of sexual harassment incident?**
